# Supplementary figures and images for: Determinants of life satisfaction among Ghanaians aged 15 to 49 years: A further analysis of the 2017/2018 Multiple Cluster Indicator Survey
Source: PLoS One. 2022 Jan 21;17(1):e0261164. doi: 10.1371/journal.pone.0261164 (PMC8782464; doi:10.1371/journal.pone.0261164)

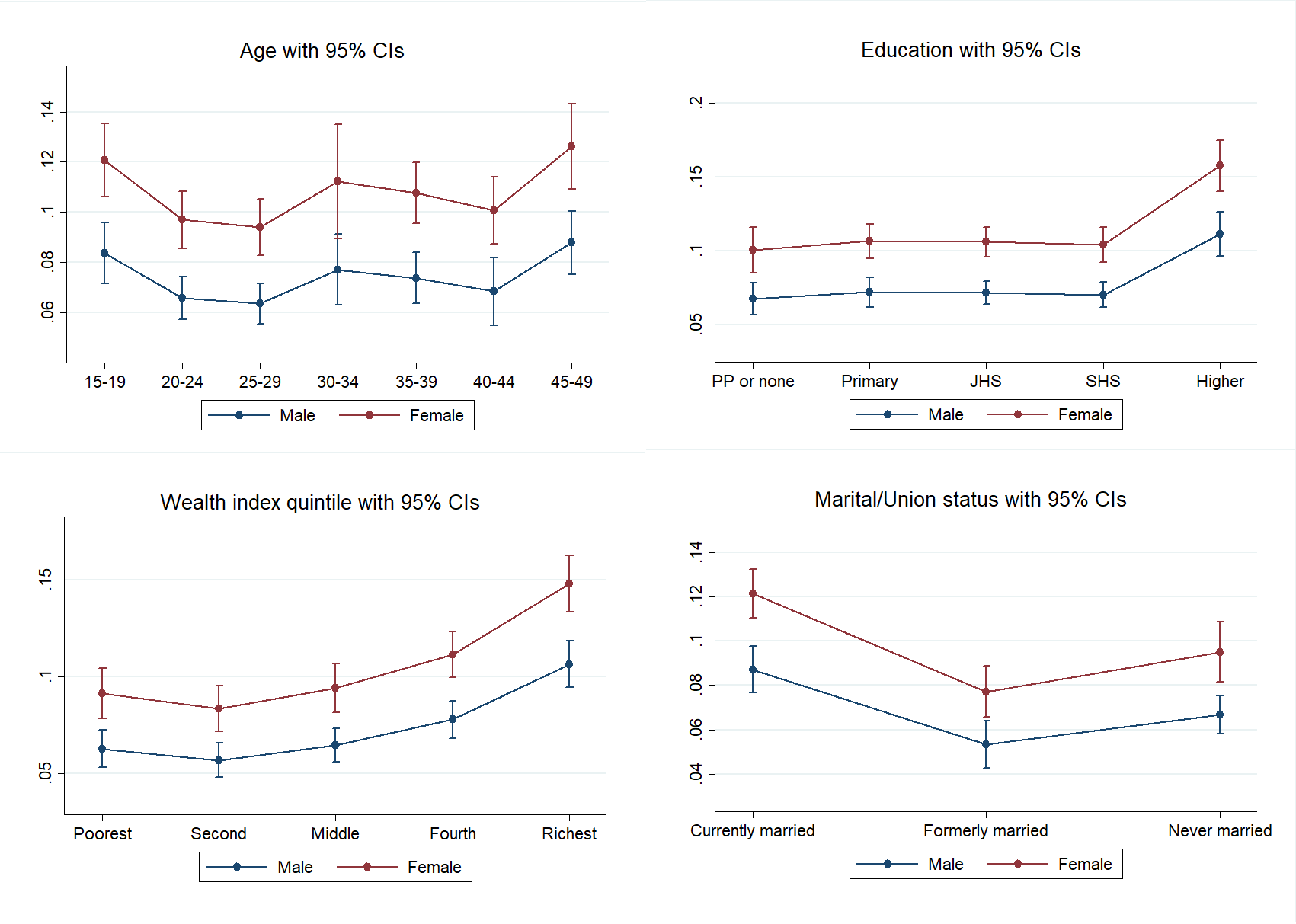

Supplement: S1 Fig — Margins plot with confidence intervals. Blue lines represent men and red lines represent women. Note: PP = Pre-primary education; predicted probabilities (on the y-axis) were derived from full sample model interacting with gender with age groups, education level, wealth quintile and marital status (each on the x-axis). (TIF) [file pone.0261164.s001.tif]
