# Supplementary material for: Determinants of life satisfaction among Ghanaians aged 15 to 49 years: A further analysis of the 2017/2018 Multiple Cluster Indicator Survey
Source: PLoS One. 2022 Jan 21;17(1):e0261164. doi: 10.1371/journal.pone.0261164 (PMC8782464; doi:10.1371/journal.pone.0261164)
Supplement: S1 Table — (PDF) [file pone.0261164.s002.pdf]

S1 Table. Intercorrelation between study variables

Variables	1	2	3	4	5	6	7	8	9	10
1. Life satisfaction	—									
2. Gender	0.09***	—								
3. Age	0.02**	0.05***	—							
4. Education	0.04***	-0.12***	-0.26***	—						
5. Marital Status	-0.04***	-0.11***	-0.67***	0.33***	—					
6. Parity	-0.00	0.16***	0.79***	-0.44***	-0.77***	—				
7. Insurance	-0.08***	-0.12***	0.01	-0.13***	0.02***	0.02***	—			
8. Household wealth index	0.07***	0.03***	0.05***	0.50***	0.08***	-0.14***	-0.15***	—		
9. Rural-urban	-0.05***	-0.04***	-0.02***	-0.34***	-0.11***	0.12***	0.10***	-0.61***	—	
10. Region of residence	0.07***	-0.03***	-0.01	-0.24***	-0.05***	0.04***	-0.08***	-0.41***	0.24***	—

Note. * $p < 0.05$, ** $p < 0.01$, *** $p < 0.001$
