# Supplementary material for: Determinants of life satisfaction among Ghanaians aged 15 to 49 years: A further analysis of the 2017/2018 Multiple Cluster Indicator Survey
Source: PLoS One. 2022 Jan 21;17(1):e0261164. doi: 10.1371/journal.pone.0261164 (PMC8782464; doi:10.1371/journal.pone.0261164)
Supplement: S2 Table — (PDF) [file pone.0261164.s003.pdf]

S2 Table. Bivariate oprobit model regressing life satisfaction on predictor variables in the full sample and gender stratified sample

	Full Sample	Male sample	Female sample
	**Coef. [95% CI]**	**Coef. [95% CI]**	**Coef. [95% CI]**
**Gender**			
Male	[ref]	NA	NA
Female	0.20*** [0.14, 0.27]		
**Age**			
15-19 years	-0.07 [-0.15, 0.003]	-0.06 [-0.19, 0.08]	-0.07 [-0.16, 0.02]
20-24 years	-0.16*** [-0.24, -0.09]	-0.23** [-0.37, -0.08]	-0.14** [-0.23, -0.05]
25-29 years	-0.12** [-0.20, -0.04]	-0.22** [-0.38, -0.06]	-0.11* [-0.21, -0.01]
30-34 years	-0.02 [-0.11, 0.07]	-0.10 [-0.25, 0.04]	-0.004 [-0.10, 0.10]
35-39 years	-0.07 [-0.15, 0.02]	-0.11 [-0.31, 0.10]	-0.06 [-0.15, 0.02]
40-44 years	-0.11* [-0.22, -0.005]	-0.04 [-0.20, 0.12]	-0.14* [-0.26, -0.01]
45-49 years	[ref]	[ref]	[ref]
**Education**			
Pre-primary or none	[ref]	[ref]	[ref]
Primary	-0.04 [-0.13, 0.05]	-0.06 [-0.29, 0.16]	-0.03 [-0.15, 0.09]
Junior Secondary	-0.05 [-0.13, 0.02]	-0.02 [-0.22, 0.17]	-0.04 [-0.12, 0.05]
Senior Secondary	-0.01 [-0.10, 0.07]	-0.04 [-0.23, 0.15]	0.06 [-0.03, 0.15]
Higher	0.34*** [0.25, 0.43]	0.38** [0.14, 0.62]	0.39*** [0.26, 0.52]
**Marital Status**			
Currently married/in union	0.30*** [0.22, 0.38]	0.54** [0.17, 0.91]	0.30*** [0.22, 0.37]
Formerly married/in union	[ref]	[ref]	[ref]
Never married/in union	0.22*** [0.15, 0.30]	0.52** [0.18, 0.85]	0.23*** [0.15, 0.31]
**Parity**			
No child	[ref]	[ref]	[ref]
One child	-0.009 [-0.09, 0.07]	-0.11 [-0.26, 0.05]	-0.05 [-0.13, 0.03]
Two children	-0.007 [-0.07, 0.06]	0.01 [-0.16, 0.17]	-0.07 [-0.14, 0.001]
Three children	0.02 [-0.06, 0.09]	0.03 [-0.11, 0.17]	-0.04 [-0.12, 0.04]
Four children	0.005 [-0.05, 0.06]	0.03 [-0.06, 0.11]	-0.06 [-0.12, 0.01]
**Insurance coverage**			
Without insurance	0.16*** [0.11, 0.21]	0.27*** [0.18, 0.37]	0.09*** [0.04, 0.14]
With insurance	[ref]	[ref]	[ref]
**Household wealth**			
Poorest	[ref]	[ref]	[ref]
Second	-0.13** [-0.22, -0.04]	-0.19* [-0.34, -0.04]	-0.12* [-0.23, -0.02]
Middle	-0.10** [-0.18, -0.03]	-0.25*** [-0.40, -0.10]	-0.05 [-0.14, 0.04]
Fourth	-0.01 [-0.10, 0.07]	-0.11 [-0.26, 0.04]	0.02 [-0.07, 0.11]
Richest	0.23*** [0.16, 0.31]	0.24*** [0.12, 0.37]	0.22*** [0.14, 0.31]
**Rural-Urban**			
Rural	0.10** [0.03, 0.17]	0.07 [-0.08, 0.21]	0.11** [0.04, 0.17]
Urban	[ref]	[ref]	[ref]

**Region of residence**			
Western	[ref]	[ref]	[ref]
Central	-0.01 [-0.13, 0.11]	-0.32* [-0.57, -0.07]	0.08 [-0.05, 0.21]
Greater Accra	0.31*** [0.21, 0.41]	0.37*** [0.16, 0.59]	0.29*** [0.17, 0.42]
Volta	0.25** [0.10, 0.40]	0.22 [-0.01, 0.43]	0.27** [0.09, 0.45]
Eastern	0.11 [-0.001, 0.23]	-0.08 [-0.30, 0.13]	0.19** [0.05, 0.33]
Ashanti	0.05 [-0.08, 0.17]	-0.03 [-0.30, 0.25]	0.07 [-0.05, 0.20]
Brong Ahafo	0.21*** [0.10, 0.32]	0.08 [-0.14, 0.30]	0.26*** [0.12, 0.39]
Northern	0.28*** [0.16, 0.41]	0.24 [-0.01, 0.49]	0.30*** [0.17, 0.44]
Upper East	0.45*** [0.32, 0.59]	0.10 [-0.17, 0.36]	0.60*** [0.45, 0.75]
Upper West	0.34*** [0.20, 0.48]	0.31 [-0.01, 0.63]	0.35*** [0.21, 0.49]

Note. * $p < 0.05$, ** $p < 0.01$, *** $p < 0.001$; 95% CI: Confidence interval; Coef.: Robust regression coefficient
