# Supplementary material for: Determinants of life satisfaction among Ghanaians aged 15 to 49 years: A further analysis of the 2017/2018 Multiple Cluster Indicator Survey
Source: PLoS One. 2022 Jan 21;17(1):e0261164. doi: 10.1371/journal.pone.0261164 (PMC8782464; doi:10.1371/journal.pone.0261164)
Supplement: Attachment — Submitted filename: Response to Reviewers.docx [file pone.0261164.s006.docx]

Response to Reviewers’ Comments

Manuscript Number: PONE-D-20-37979

Dear Editor,

Thank you for your letter and the opportunity to revise our paper on “Determinants of life satisfaction among Ghanaians aged 15 to 49 years: Further analysis of the 2017/2018 Multiple Cluster Indicator Survey”. We are grateful for the reviews provided by the reviewers.

Thank you for your continued interest in our research and we hope that this improved manuscript is accepted for publication in PLOS ONE. Below are our responses to the comments and concerns.

Editor comments

1. Please ensure that your manuscript meets PLOS ONE's style requirements, including those for file naming. The PLOS ONE style templates can be found at

<https://journals.plos.org/plosone/s/file?id=wjVg/PLOSOne_formatting_sample_main_body.pdf> and

https://journals.plos.org/plosone/s/file?id=ba62/PLOSOne_formatting_sample_title_authors_affiliations.pdf

Response

Thank you for the reminder. We have followed the formatting requirements accordingly.

2. In the methods section, please provide details regarding how the household wealth index was categorized

Response

The authors have provided these details. “*Household wealth was categorized into poorest (0), second quintile (1), middle (2), fourth quintile (3), and richest (4).**”*

Reviewer comments:    
Reviewer #1:

1. inclusion of happiness as a predictor: I believe that including happiness as a predictor will bias all your estimates. Why is that? First, think about what “the effect of X on life satisfaction, controlling for happiness” ought to mean – happiness is extremely closely related to life satisfaction. What does it mean to be more satisfied without being happier? More rigorously, I believe that a sensible model would be that both happiness and life satisfaction are measures of people’s underlying overall assessment of their lives. Controlling for happiness will remove a lot of valid variability. Thinking about it another way: how satisfied you are with your life may affect how happy you are right now. Happiness is thus an outcome of life satisfaction, including it in a model will introduce collider bias. I’ve written about collider bias elsewhere, maybe you’ll find it helpful: Rohrer, 2018, <https://journals.sagepub.com/doi/full/10.1177/2515245917745629>. I’d consider any of the following solutions satisfactory: omit happiness as a predictor; combine happiness and life satisfaction into a more reliable indicator of well-being (see also point below on dichotomization of the outcome); analyze both as outcomes separately (as robustness check, but also maybe to find out whether these measures react differently to various predictors).

**Response**

Thank you for noting these errors. As suggested, we have omitted happiness as a predictor.

2. Dichotomization of the outcome: I’m opposed to the dichotomization of the outcome; I believe that this steps discards information that could be valuable. I guess there could be some concern that respondents are not fully using the scale in a “reasonable” manner. That may as well be possible (I think in representative samples, many people are overwhelmed by too many response options), but dichotomizing isn’t really going to fix this issue—it just imposes the assumption that all 0-6 responses are the same, and that all 7-10 responses are the same, discarding valuable information (I do believe that somebody who says 0 is likely less satisfied than somebody saying 6, but your analysis would discard this information). If people don’t use a scale efficiently, they may as well “accidentally” respond 7 instead of 6, so you still have misclassification.
   There is another issue with analyzing a dichotomous outcome: You know have to decide on which scale to assess interaction. Currently, you’re only looking at Odds Ratios. I believe that many economists wouldn’t be satisfied with that, as they are very much in favor of evaluating interactions on a probability scale. As it happens, I just had a paper accepted covering this topic, you may find it helpful: Rohrer & Arslan (2021), preprint: <https://psyarxiv.com/7fm2j/> (relevant section is the first one on the scale dependence of interactions). If you keep the binary outcome, I’d like to see an evaluation of interaction on the scale of the probability of being thriving (Odds Ratio is rather unintuitive, its only merit are some nice statistical properties). If you instead do a regular linear regression, that won’t be an issue. (I’m aware that doing a simple linear regression with an ordinal output isn’t optimal either; but I think the proper solution would be an ordinal model rather than dichotomization. But I do think ordinal models are rather involved and often not quite interpretable, so I wouldn’t want you to run one of those either).

**Response**

Thank you for this suggestion. We have removed dichotomization of the outcome variable. Currently, we are using the variable in its original state, using all the points on the Cantril’s Self-Anchoring Ladder of Life Satisfaction scale. Correspondingly, the data analysis was changed from binary logistic regression to ordered probit regression as you suggested.

3. abstract, “in a full and gender-stratified model”: when reading this for the first time, I was really confused what “full” was referring to. A “full model”? A “fully-stratified” model? I assume you mean to say that you did analysis in two ways, one time for the full sample, one time for gender subsamples

**Response**

This correction has been done. Kindly find this correction on the abstract page. “*This study, therefore, extends previous literature by examining the determinants of life satisfaction among Ghanaians in two ways: a full sample and gender-stratified sample.”*

4. line 74, “the age of Addai’s et al. dataset was within 2005-2008 while Calys-Tagoe’s et al. research focused on older adults 50years and above.”: I don’t understand what is being said here (is this a comparison of dates with ages?)

**Response**

This correction has been done. Kindly find this correction on page 4. *“The age of Addai et al’s (41) dataset is now quite old, using data that was collected within 2005-2008. Also, Calys-Tagoe et al’ study (12) only focused on older adults (50 years and above).”*

5. p. 8, bivariate analysis: I don’t think it’s a good idea to use significance in a bivariate analysis as a criterion for inclusion of a variable. I even think I have seen people writing about this (it’s prone to overfitting, and in any case a bivariate association doesn’t tell you whether a variable has a causal effect or not). I still think the bivariate analysis is nice for full transparency, so I’d simply delete the sentence using it as a rationale for inclusion in the full model.

**Response**

Thank you for this suggestion. We have removed this sentence from the manuscript.

6. - I do like that you report gender-stratified analyses

**Response**

Thank you for the feedback.

Reviewer #2:

1. Most importantly, the theoretical focus of the paper needs to be clarified (p. 3-5). To illustrate, the authors state the importance of studying life satisfaction in Ghana and list a number of potential underlying factors such as poverty, but don’t elaborate on potential underlying mechanisms and how all of these constructs are linked to one another. It would be very helpful to also conceptualize these theoretical assumptions in the study or reframe the introduction so that it becomes clear how life satisfaction and the covariates are linked to one another, what the role of the specific regional context is and how this can be embedded into the existing literature

**Response**

Thank you for this suggestion. We have accordingly reframed the introduction by clearly indicate how life satisfaction and the covariates are linked to one another. Here is an example of text elaborating this on page 3. *“In Diego-Rosell, Tortora, and Bird’s (35) study, for instance, it was found out that families with a high household income had better life satisfaction across the 153 countries. Luhmann et al. (36) also found from analysing longitudinal data from three nationally representative panel studies that life satisfaction levels increased with marriage and childbirth but reduced with marital separation, job loss, starting a new job, and relocating”.*

2. The literature review is incomplete. To illustrate, a number of studies have examined life satisfaction in adults (e.g., Blanchflower & Oswald, 2008; Diener, Suh, Lucas, & Smith, 1999; Pavot & Diener, 1993). Many of these studies have also examined the role of individual difference characteristics for life satisfaction (e.g., living status). Some of those studies in a longitudinal manner. It is thus necessary to further highlight what the present study adds to the existing literature. Please clarify.

**Response**

Thank you for bringing this to our attention. We have further highlighted on how this present study adds to existing literature. See page 5, *“Our study goes a step further by examining closely these factors from a gendered perspective; an examination that is virtually nonexistent in Ghana. Due to the variations in social norms and biological characteristics, a gendered perspective has been recommended to provide more nuanced information into the associated factors of both men and women’s life satisfaction (36–38).”*

3. p. 5-6: I stumbled across the sample selection and study description. To illustrate, it should be clearer what the recruitment strategy for the sample was. For example, are people of the same household part of the sample? If so, data wouldn’t be independent from one another and the model would need to account for potential statistical interdependence. Alternative one would need to ensure to use only one member of the household.

**Response**

Thank you for this suggestion. We have clearly indicated the recruitment strategy for the sample. A complex survey design was used to account for statistical interdependence. See page 9, *“In each household, people from the same household were selected.”* and, page 6, *“We accounted for the complex sampling design embedded in the dataset to monitor possible analytical errors and make proper inferences (45). This was achieved by correcting for clusters, stratification, and sample weights using the complex survey mode command 'svyset'.”*

4. p. 8: The authors should provide an intercorrelation table of all variables under study. This is important because it could provide further insight into the association of their related but ideally distinct outcome measure.

**Response**

Thank you for bringing this to our attention. An intercorrelation table has been included as supplementary material. For easy identification of the intercorrelation table, a statement has been provided. See page 12, *“Spearman’s rho was used as a preliminary test to assess the intercorrelations between all study variables (see S1 Table).”*

5. p. 8: Relatedly, I was surprised to see that the authors controlled for happiness when examining life satisfaction. From a conceptual perspective, both constructs are closely related to one another and might even be used interchangeably, depending on the measure that is used. I would also expect the constructs to be highly corrected. If not, this would hint at the measures examining something different but if so, this would result in multicollinearity issues

**Response**

Thank you for noting this. As suggested, we have omitted happiness as a predictor.

6. p. 8: My biggest concern is that the authors dichotomized the outcome variable (struggling versus thriving) and applied logistic multiple regression analyses. In general, one should always make use of all data available. Artificially dichotomizing variables will result in a loss of information that would have been available otherwise. The authors have to make use of all data available by running at least a multiple regression analysis or ideally examine all variables multivariate framework (e.g., SEM). Please clarify.

**Response**

Thank you for this suggestion. We have removed dichotomization of the outcome variable. Currently, we are using the variable in its original state, using all the points on the Cantril’s Self-Anchoring Ladder of Life Satisfaction scale. Correspondingly, the data analysis was changed from binary logistic regression to ordered probit regression as you suggested.

7. p. 8: Relatedly, did the author test for any interaction effects? To illustrate, it could be that life satisfaction varies for older men but not older women (i.e., age-gender interaction) or for lower educated individuals living alone but high educated individuals living alone. Please clarify.

**Response**

There was no interaction effect tested in the new analysis. Although, the authors acknowledge the impact of such interaction, testing interaction effect was beyond the scope of the study’s objectives. Your suggestion has been recommended for future researchers. See page 21, “*Additionally, future Ghanaian studies should attempt using other robust analysis such as multilevel modelling as well as testing interaction effects (e.g., age-gender interaction).”*

8. p. 9: Reporting percentages is not very informative. When examining a multivariate regression using continuous outcomes and predictors, the authors would need to report the regression coefficient and also effect sizes.

**Response**

We have reported the regression coefficient and average marginal effect. These can be found in Table 2 and 3.

9. p. 9: Data on the geographical regions is part of the dataset. This is very interesting and could be of potential use for the authors. However, study participants are then nested in geographical regions and thus multilevel modeling should be applied.

**Response**

Thank you for this suggestion. Although we agree that multilevel modeling is one of the alternative methods, complex survey analysis also accounts for these inherent variations and clusters. We, therefore, have recommended the use of multilevel modelling for future studies in Ghana. See page 21, *“Additionally, future Ghanaian studies should attempt using other robust analysis such as multilevel modelling as well as testing interaction effects (e.g., age-gender interaction).”*

10. p. 1: The authors switch between the terms well-being and life satisfaction throughout the manuscript. I would suggest sticking to one and provide a definition upfront.

**Response**

We have accordingly sticked to the use of only life satisfaction in this manuscript. You can find these throughout the manuscript.

11. p. 8: Were variables of interest standardized or centered (e.g., mean-centered). Please clarify.

**Response**

The analysis was redone using ordered probit regression. Because ordered probit regression accounts for all the points in the outcome variable, there was no need for us to standardize or center the variable.

12. e.g., p. 17: Throughout the manuscript, the authors imply directionality and sometimes causality in the associations between life satisfaction and the chosen covariates. I would encourage the authors to avoid any causal or directional language since the analysis does not allow to make any conclusions about causality or directionality.

**Response**

Thank you for noting this. We have accordingly removed any causal language. Here is an example of text highlighting this change, on page 16, “*The association between marital status, parity, household wealth index, region of residence, and life satisfaction were different for females and males.”*

13. p. 4: relatedly, since the data is cross-sectional the study does not examine ‘factors which contribute to the maintenance of a high level of life satisfaction in Ghana’ but ‘factors which are associated with life satisfaction in Ghana’.

**Response**

Thank you for suggesting this. We have reframed our introduction and thus, this statement has been omitted from the work.

14. Title, Abstract, p. 1-21: I wonder if it is necessary to add the term reproductive to the sample description when not discussing it further. E.g., would the authors expect this result to be an effect of reproductivity? Also, can one compare the reproductive age of men and women?

**Response**

This correction has been made. Kindly find this correction on the abstract page “*This study, therefore, extends previous literature by examining the determinants of life satisfaction among Ghanaians in two ways: a full model and gender-stratified model”* and the title page, “*Determinants of life satisfaction among Ghanaians aged 15 to 49 years: A further analysis of the 2017/2018 Multiple Cluster Indicator Survey.”*
