# Supplementary material for: Determinants of life satisfaction among Ghanaians aged 15 to 49 years: A further analysis of the 2017/2018 Multiple Cluster Indicator Survey
Source: PLoS One. 2022 Jan 21;17(1):e0261164. doi: 10.1371/journal.pone.0261164 (PMC8782464; doi:10.1371/journal.pone.0261164)
Supplement: Attachment — Submitted filename: Response to Reviewers.docx [file pone.0261164.s007.docx]

Dear Dr. José Antonio Ortega,

Thank you very much for giving us another opportunity to improve our manuscript. Below are the responses to the editorial and reviewer’s comments.

**Editor’s review**
There is sometimes a choice of innacurate words like rudimentary for elemmentary.

**Authors’ response**

We have rectified this error. The new sentence reads like this: *“Briefly, most of the selected variables were measured in a simple manner…”*

**Editor’s review**

The paper is missing a methods section describing the ordered probit model used explaining its strengths and limitations in this context.

**Authors’ response**

Thank you for noting this omission. We have updated the manuscript accordingly. Find this on page 8: *“The ordered probit model is typically used to examine the variation in the data points of an ordinal categorical dependent variable (44). Though argued to produce parameter estimates difficult to interpret, oprobit was fitted mainly for its ability to preserve the ordering of the response options in the outcome variable as a function of the predictor variables (45).”*


**Journal Requirements:**

Please review your reference list to ensure that it is complete and correct. If you have cited papers that have been retracted, please include the rationale for doing so in the manuscript text, or remove these references and replace them with relevant current references. Any changes to the reference list should be mentioned in the rebuttal letter that accompanies your revised manuscript. If you need to cite a retracted article, indicate the article’s retracted status in the References list and also include a citation and full reference for the retraction notice.

**Authors’ response.**

The reference list has been double checked and we found no retracted papers. However, three more references were included in the reference list.

**Reviewers' comment**

Reviewer #1: I’d like the authors for carefully addressing all of my comments. My previous concerns no longer apply to the revised version of the manuscript, which I believe will make a great contribution to the literature on well-being. I have only very few minor remarks left, such as typos or small tweaks to the language. Apart from that, I’d like to encourage the others to make their Stata do-files available for the scientific community, for example, on the Open Science Framework ([osf.io](http://osf.io/)). That way, other researchers can apply for access to the data used and then use the do-files to reproduce results; but it is also really helpful to double-check how exactly models were specified.
(I believe that this would also be aligned with PLOS ONE’s Data Availability Policy)

**Authors’ response**

Thank you for the feedback and yes, the Stata do-file as well as the dataset are attached to the revised manuscript.

**Reviewer’s comment**
p. 3, “life satisfaction across the 153 countries”: I think this should either be “across 153 countries” or “across the 153 countries included in their study.”

**Authors’ response**

Thank you for pointing this out. We have accordingly corrected this sentence: *“…it was found that families with a high household income had better life satisfaction across 153 countries.”*

**Reviewer’s comment**
p. 4, “Considering these limitations and the pressing need to produce more recent evidence regardless of the persisting contextual problems ranging from limited access to drinking water [26], unemployment particularly among the youth [27], limited access to health care, poverty [28], high prevalence of chronic diseases [29–33] and poor quality education [34,35] that may threaten one’s life satisfaction, our study used the 2017/2018 Multiple Cluster Indicator Survey to examine the factors associated with life satisfaction in Ghana.”: I find the usage of “regardless of” here confusing. I wouldn’t say we need more evidence regardless of the problems, I would say we need more evidence in particular because of these problems?

**Authors response**

Thank you for noting this error and the suggestion. The sentence has been restructured as such: *“Considering these limitations and the pressing need to produce more recent evidence in particular because of persisting contextual problems ranging…”*

**Reviewer’s comment**
p. 6, “The final samples were 660 clusters and 13202 households to the sampling strata.”: I’m confused by “to the sampling strata”; do you mean “across all sampling strata”?

**Authors’ response**

Indeed, we meant “across all sampling strata” and this correction has been made. Thank you.

**Reviewer’s comment**
p. 7, “This recategorized variable was kept for only descriptive purposes; therefore, the original variable (i.e., the 0 to 10 ordinal variable) was used in the main study analyses.”: I think this sentence actually becomes clearer if you just omit the “therefore”

**Authors’ response**

This error has been corrected thanks to your suggestion. Here is the updated sentence: *“This recategorized variable was kept solely for the purposes of description; the original variable…”*

**Reviewer’s comment**
p. 15, Table 3, 2 column from left, Parity Effects for Men at middle life satisfaction: there’s five cells with the same value (.195), I suspect this may be a copy-paste-error

**Authors’ response**

Interestingly, this is not a copy-paste error. Similar values were generated through the margins command and rounded to .195. We are assuming they are the same because the probabilities are being predicted at the middle level of life satisfaction.
